# The human transmembrane proteome

**DOI:** 10.1186/s13062-015-0061-x

**Published:** 2015-05-28

**Authors:** László Dobson, István Reményi, Gábor E. Tusnády

**Affiliations:** “Momentum” Membrane Protein Bioinformatics Research Group, Institute of Enzymology, RCNS, HAS, Budapest, PO Box 7, H-1518, Hungary

**Keywords:** Transmembrane protein, Topology prediction, Hidden markov model, Constrained prediction

## Abstract

**Background:**

Transmembrane proteins have important roles in cells, as they are involved in energy production, signal transduction, cell-cell interaction, cell-cell communication and more. In human cells, they are frequently targets for pharmaceuticals; therefore, knowledge about their properties and structure is crucial. Topology of transmembrane proteins provide a low resolution structural information, which can be a starting point for either laboratory experiments or modelling their 3D structures.

**Results:**

Here, we present a database of the human α-helical transmembrane proteome, including the predicted and/or experimentally established topology of each transmembrane protein, together with the reliability of the prediction. In order to distinguish transmembrane proteins in the proteome as well as for topology prediction, we used a newly developed consensus method (CCTOP) that incorporates recent state of the art methods, with tested accuracies on a novel human benchmark protein set. CCTOP utilizes all available structure and topology data as well as bioinformatical evidences for topology prediction in a probabilistic framework provided by the hidden Markov model. This method shows the highest accuracy (98.5 % for discrinimating between transmembrane and non-transmembrane proteins and 84 % for per protein topology prediction) among the dozen tested topology prediction methods. Analysis of the human proteome with the CCTOP indicates that it contains 4998 (26 %) transmembrane proteins. Besides predicting topology, reliability of the predictions is estimated as well, and it is demonstrated that the per protein prediction accuracies of more than 60 % of the predictions are over 98 % on the benchmark sets and most probably on the predicted human transmembrane proteome too.

**Conclusions:**

Here, we present the most accurate prediction of the human transmembrane proteome together with the experimental topology data. These data, as well as various statistics about the human transmembrane proteins and their topologies can be downloaded from and can be visualized at the website of the human transmembrane proteome (http://htp.enzim.hu).

**Reviewers:**

This article was reviewed by Dr. Sandor Pongor, Dr. Michael Galperin and Dr. Pascale Gaudet (nominated by Dr Michael Galperin).

**Electronic supplementary material:**

The online version of this article (doi:10.1186/s13062-015-0061-x) contains supplementary material, which is available to authorized users.

## Background

The biological functions of transmembrane proteins (TMPs) are widespread. They are involved in diverse biological processes ranging from basic and primordial life functions such as energy production to the most advanced molecular functions of a multicellular organism, e.g. cell-cell communication or synaptic transmission. Despite these important roles, there are only about a hundred human TMPs with experimentally determined 3D structure [1–3]. Due to the difficulties inherent to membrane protein crystallography and the currently available rather laborious experimental methods for topology determination, only several thousands of experimentally established topology data are available [4]. Thus, computational approaches for predicting the 3D structure of TMPs are highly required.

After completing the Human Genome Project [5, 6], it was determined that about 25–30 % of the coded protein in the human genome encode TMPs. This means that there are 6–8 thousand TMPs in the human proteome. Although the reported per protein transmembrane topology prediction accuracies of the various algorithms (see Additional file 1) were shown to be above 80 %, they reached rather low prediction accuracies on a human benchmark set (see below). This may be because the topology prediction algorithms were trained and tested on benchmark sets containing mostly prokaryotic TMPs, whose properties (e.g. amino acid composition, local structure) differ from the properties of eukaryotic and thus human TMPs. The accurate prediction of the topology is a crucial step in the 3D structure prediction process, since most 3D prediction algorithms start from the predicted topologies. It is obvious that relying on a wrong topology model will result in an inappropriate 3D structure, like in the case of the proposed structure of the human ABCG2 protein [7].

Predicting the presence of signal peptides before topology prediction is important but often neglected. Detailed analysis revealed that topology prediction methods often mistake signal peptides for transmembrane helices (TMHs) due to their similar physical-chemical properties [8, 9]. Signal peptides control proper targeting of proteins which are destined toward the secretory pathway. Signal peptides are located at the N-terminus of proteins and contain a hydrophobic region, which is very similar to the TMHs both in length and in amino acid composition [10, 11]. Cleavable signal peptides can be identified by simple statistical means [12, 13] or modern machine learning approaches such as Artificial Neural Network, Hidden Markov Model or Support Vector Machine with high sensitivity (95–98 %) and specificity (93–98 %) [10, 11, 14, 15]. The presence of a signal peptide on the N-terminus of a TMP indicates extra-cytosolic location. One approach to reduce false prediction of signal peptides as well as false prediction of TMHs is the combination of signal peptide prediction and transmembrane topology prediction [8, 16, 17]. However, signal peptide prediction and topology prediction are two different tasks, therefore removing signal peptides from the amino acid sequences before topology prediction is a better approach [18, 19].

In addition to the computational approaches, there are several high throughput biotechnological methods, which were utilized to characterize transmembrane proteins on a genomic scale. Investigation of the cell surface proteome by means of biotinylation of surface proteins and purifying the marked proteins by affinity chromatography on avidin agarose resin and analyzing by SDS-PAGE followed by liquid-chromatography mass spectrometry (LC-MS/MS) became a routine task in molecular biology laboratories. This was used for the analysis of the protozoan *Trichomonas vaginalis* surface proteome [20], for the investigation of the *Entamoeba histolytica* surface proteome [21], and for the characterization of several human cell lines including embryonic stem cells [22] and human mesenchymal stromal cells [23]. Nowadays it is commonly used to identify transmembrane proteins in various types of cancer in order to develop new antibody-based therapies [24].

This high-throughput technique is useful in gene annotations, but does not produce usable topology information about the TMPs. However, if the protein is glycosylated, the results of high-throughput techniques can indicate the localization of a certain part of the amino acid sequence. Glycosylation is the most common post-translational modification of TMPs and extracellular proteins, in which the side chain of Asp (N-Glycosylation) or of Ser/Thr/Trp (O-Glycosylation) is modified by the attachment of a sugar component. These modifications occur only at the extra-cytosolic side of proteins, therefore knowledge about the sequential localization of these modifications presents topological information about TMPs as well. Incorporating this information and other experimentally established topological data into prediction methods can highly increase the accuracy of the prediction [4, 25–28]. The extent of this increase was estimated to be at least 10% in whole-genome predictions using TMHMM algorithm [29], constrained by limited experimental information (such as the in/out location of a protein’s C-terminus) [30].

In addition to the incorporation of experimental data into the prediction methods, other information generated by computational methods can also be used as constraints. For example, protein domains and sequence motifs, which are conservatively located on the cytoplasmic or extra-cytoplasmic side of the membrane. Such domains and motifs can be generated by merging the data of the various domain and motif databases with the topology information and may be used in the same manner as the results of topology experiments. Such a combination of domain/motif data with topology data were used during the creation of the TOPDOM database [31] by a fully automated algorithm. It was also shown, by identifying a set of 367 domains from soluble proteins in the SMART database which have compartment-specific localization of a type relevant to membrane protein topology prediction, that high-quality topology models can be provided utilizing these domains as prediction constraints, for 11 % of the membrane proteins extracted from 38 eukaryotic genomes [32].

Estimating the reliability of the prediction methods is an important issue. There were several early attempts to determine the reliability of topology prediction methods on a genomic scale, which we could not directly access before due to the unavailability of high throughput experimental topology data. Investigation of a subset of the *Escherichia coli* genome [33] resulted in an interesting observation, namely that the reliability of TM segment prediction correlates positively with the number of prediction methods producing the same topology. Based on these findings, Käll and Sonnhammer (2002) [34] developed a consensus prediction method to estimate the reliability of the TM prediction on whole genome data by counting how many methods agree in their consensus predictions. However, their results can be interpreted that the five prediction methods agreed more on the benchmark set than the various genomes. According to our view, this indicates that these methods were trained and tested on similar benchmark sets, that show high similarity to the small prokaryotic genomes regarding the TMPs. Accordingly, the agreement of these methods should correlate to how similar is the data set (e.g. a genome) to the benchmark set, rather than to the reliability of the prediction accuracies of the methods. The problem of the overestimation of the accuracy of a prediction on small prokaryote benchmark sets has also been reported by others [30]. In the mentioned work, the reliability scores constructed for the TMHMM algorithm [29] were tested using prokaryote, and eukaryote whole genome data. The authors found that the available test set is biased towards high-scoring proteins when compared to genome-wide data sets.

Here we report the Human Transmembrane Protein (HTP) database containing structural information on human α-helical TMPs. Structural information is classified into several evidence levels. The prediction accuracy of the CCTOP consensus method used for creating the database is the highest on a newly established benchmark set containing more than 450 human transmembrane proteins. We propose a novel algorithm as well to distinguish between globular and TMPs using their amino acid sequence only. This filtering algorithm was tested on an assembled set consisting of globular and human transmembrane proteins. We also suggest a way to avoid the problem of misprediction of signal peptides as transmembrane helices. In addition to these proposed algorithms, we collected the results of high throughput glycosylation sequence data in a recent TOPDB update and used them together with the topology data collected so far in the PDBTM, TOPDB, and TOPDOM databases. We made the HTP database available on the internet to download and to investigate 3D structure and/or topology data of TMPs online at http://htp.enzim.hu.

## Methods

### Databases used

The human proteome has been downloaded from UniProt [35] (UniRef 90 Human Proteome) in March, 2013. It comprises 19,584 sequences. In the current version of the HTP database, we do not use the alternatively spliced protein sequences.

Topology data were collected from three different resources: the PDBTM (31/05/13), the TOPDB (version 1), and the TOPDOM (version 1.089) databases. The most reliable data can be found in the PDBTM database [1–3], which contains the 3D structure of TMPs together with the most likely membrane orientation determined by the TMDET algorithm [36]. Because PDBTM does not contain topology information, only the sequential localizations of the TMHs, topology information was collected from the TOPDB database [4].

The TOPDB database [4] was established 6 years ago, containing the experimentally determined topology data of TMPs. The initial database contains 23,164 topology data from about 1500 TMPs. We have recently updated TOPDB from several sources. These were i) topography information defined by the TMDET algorithm using the 3D structure from PDBTM database, extended by topology information from articles containing the description of the original 3D structures; ii) experimental data published in the last couple of years; iii) global topology analysis of yeast [37]; iv) topology data generated by high throughput techniques, like the sequential positions of N- or O- glycosylations. More than 41,000 new topology data and almost 2000 new TMPs have been collected, and now TOPDB contains more than 65,000 topology data of 3436 TMPs.

The third resource was TOPDOM [31]. TOPDOM is a collection of domains and sequence motifs located conservatively on the cytosolic or extra-cytosolic side of TMPs.

### Preparation of the benchmark data sets

The TOPDB database was split into two parts; the first contains entries with known 3D structures, while the second set contains entries with topologies confirmed only by molecular biology experiments. Entries whose reliability is above 99 and 95 % for bitopic and polytopic transmembrane proteins, respectively, were selected. For each sequence in the human proteome, BLAST searching was performed against these two sets. The resulting hits were aligned with the query sequences using high-scoring segment pairs (HSPs), and those were kept, which i) had a sequence identity above 40 %, ii) the overlapping sequences covered all TM helices of the TOPDB entry, and iii) the length of the hit sequence was above 80 % of the length of the query sequence. Finally, we filtered these sets by the CD-HIT algorithm [38, 39] to 40 % sequence identity. This resulted in 136 sequences, which have homologous partner in the PDBTM database with known 3D structure (“3D benchmark set”), and 338 sequences, of which the homologous partners are characterized by experimental topology data only (“experimental benchmark set”).

A third benchmark set was created for testing the discriminating ability of the various methods. The merged structural and experimental benchmark set was combined with the sequences of globular proteins selected randomly from the PDBSelect database [40] in a manner that ensured that the ratio of the globular and TMP sequences will be the same as it was formerly predicted for the human genome (25 % TMP). The final filtering benchmark set contains 474 TMPs, 1422 non-TMPs, altogether 1896 sequences.

As the best filtering method resulted in 467 true positives (see Table 2), these 467 proteins (134 in 3D benchmark set and 333 in experiment benchmark set) were used to test the topology accuracies of the various prediction methods.

### Filtering transmembrane proteins

We tested eight prediction methods for their discriminating ability, i. e. the ability to determine whether a sequence codes a TMP or a non-TMP. These methods are MEMSAT-SVM [41], Octopus [42], Philius [16], Phobius [9, 16], Pro-TMHMM [43], Scampi-single [44], Scampi-MSA [44] and TMHMM [29, 45]. These methods were run on preprocessed sequences, i.e. after the removal of transit and/or signal peptides from the query sequences. As none of these method’s accuracies were as high as desired, a simple consensus approach was utilized to increase the prediction accuracy. Dozens of combinations of these approaches and parameters were tested and the best was chosen for the final consensus algorithm. We reached the best accuracies when three out of the eight methods, namely Phobius, Scampi-single and TMHMM were used for filtering, and at least two of these three methods predicted at least one membrane region (Additional file 2).

### Constrained consensus topology prediction

We tested the accuracies of several prediction methods on the benchmark sets (Additional file 1). After the testing, ten methods were selected according to their prediction accuracies, their availability and how they can be integrated into a consensus method. We tried to select methods that were based on different algorithm types. The selected methods are: HMMTOP [25, 46], Membrain [47], MEMSAT-SVM [48], Octopus [42], Philius [16], Phobius [9], Pro-TMHMM [43], Prodiv-TMHMM [43], Scampi-MSA [44] and TMHMM [29, 45]. In a recent investigation of integral membrane channels and carrier proteins five out of these ten methods were compared, and three of them were applied in a novel program, TM-STATS, to tabulate topological predictions for any subdivision of TCDB [49].

We searched for each TMP sequence with BLAST against the TOPDB database with the parameter e-value 10^−10^. Hits were accepted if the following clauses were all true: i) the hit’s length was above 80 % of the query sequence’s length; ii) all TM helices were covered in the homologous TOPDB entry by the alignment; iii) sequence identity was above 40 % within HSPs. Topology data of the homologous proteins in the TOPDB database were used in the constrained prediction by mirroring their sequential positions according to the position of the HSPs.

The search engine of the TOPDOM website was used to locate domains/motifs in the human sequences that were found earlier conservatively on the same side of TMPs, and we used the position and topology localization of the result(s) as constraint(s).

The newly developed consensus prediction algorithm is based on the probabilistic framework provided by the hidden Markov model, therefore the HMMTOP method can be utilized for this task. Briefly, the results of the ten prediction methods together with the available 3D or experimental topology data can be applied as weighted constraints in the HMMTOP to obtain the constrained consensus prediction result. The weights depend on the per-protein topology or topography accuracies of the methods.

The results of the i^th^ method are:

1 $$ {Pred}_i={l}_1,{l}_2,\dots, {l}_n,1\le i\le m, $$

2 $$ {l}_j\in \left\{``I",``M",``O",``L",``U"\right\},1\le j\le n $$

where m ≥ 10 (the ten prediction methods and zero, one or more 3D/experimental topology constraints), n is the length of the query sequence and the “I”, “M”, “O”, “L”, “U” labels correspond to cytoplasmic loops, membrane spanning segments, non-cytoplasmic loops, membrane re-entrant loops and unknown regions, respectively.

We calculated the per-protein topography (Acc_Tpg_) and topology (Acc_Top_) accuracies of each method on the “structure benchmark set”, and used these values as weights for the constraints. These values are between 0 and 1. Acc_Tpg_ was applied for the positions where the prediction method resulted in transmembrane or re-entrant loops (label “M” or “L”, respectively), otherwise Acc_Top_ was used (for label “I” and “O”). In the case of 3D or experimental topology data, the weights were set to 20. In the case of prediction methods, the results of the given prediction were used as constraints, but only if the prediction was valid, i.e. it contains at least one transmembrane region:

3 $$ {W}_{i,j}=\left\{\begin{array}{l}Ac{c}_{Top}(i),\; if\;{Pred}_{i,j}\in \left\{``I",``O"\right\}\; and\; type(j)= prediction\; method\hfill \\ {}Ac{c}_{Tpg}(i),\; if\;{Pred}_{i,j}\in \left\{``M",``L"\right\},\; and\; type(j)= prediction\; method\hfill \\ {}20,\; if\; type(j)= experimental\; results\hfill \end{array}\right.,\kern0.36em 1\le i\le m,\;1\le j\le n, $$

These weights were normalized to one in each sequential position, and were used as constraints in the HMM, as described by Bagos et al. [50]

4 $$ {C}_{j,k}=\frac{{\displaystyle \sum_{i=1}^m{W}_{i,j}\cdot \Delta \left(k,{Pred}_{i,j}\right)}}{{\displaystyle \sum_{k=1}^N{\displaystyle \sum_{i=1}^m{W}_{i,j}\cdot \Delta \left(k,{Pred}_{i,j}\right)}}},\kern0.24em 1\le j\le n,\;1\le k\le N, $$

where N is the number of states in the hidden Markov model and

5 $$ \Delta \left(a,b\right)=\left\{\begin{array}{c}\hfill 1,\; if\; label\left({S}_a\right)=b\hfill \\ {}\hfill 0,\; if\; label\left({S}_a\right)\ne b\hfill \end{array},\kern0.24em 1\le a\le N,\;1\le b\le \widehat{N},\right. $$

where $\widehat{N}=4$, the number of the main states (inside, outside, membrane and loop) and S denote states of the hidden Markov model. If MEMSAT-SVM or Octopus methods resulted in re-entrant loop regions, or re-entrant loop regions were used as 3D or experimental topology constraints, a modified architecture for HMMTOP algorithm was used, allowing the extra “language rule” for the hidden Markov model.

### Measuring the reliability of the consensus prediction

The source code of the HMMTOP program has been modified in order to calculate the sum of the posterior probabilities along the Viterbi path. According to the unique hidden structure of the HMMTOP, the posterior probabilities were summed up for each main hidden state type (inside, membrane, loop and outside) in each position of the amino acid sequence, then these probabilities were summed up along the most probable state sequence provided by the Viterbi algorithm. We use this sum divided by the length of the protein to measure the reliability. Assuming the notations of Rabiner’s excellent tutorial on hidden Markov models [51], the posterior probabilities can be calculated from the forward and backward variables:

6 $$ {\gamma}_t(i)=\frac{\alpha_t(i)\cdot {\beta}_t(i)}{P\left(O\Big|\lambda \right)},1\le t\le n,1\le i\le N, $$

where n is the sequence length, N is the number of states in the hidden Markov model, O is the array of observation symbols (the amino acid sequence) and λ is the hidden Markov model. The posterior probability of each main state can be calculated by summing up the posterior probabilities, which have the same label as the main state:

7 $$ {\Gamma}_t(j)={\displaystyle \sum_{k=1}^N{\gamma}_t\left(k\Big| label\left({S}_k\right)=j\right)},1\le j\le \overset{\wedge }{N} $$

Reliability is the average of the posterior probabilities along the most probably state path (q):

8 $$ R=100\cdot \frac{{\displaystyle \sum_{t=1}^n{\Gamma}_t\left( label\left({q}_t\right)\right)}}{n} $$

### Generating the human transmembrane proteome database

All transit peptides have been cleaved using UniProt annotations, then the signal peptides have been predicted by SignalP 4.1 [11, 52]. This prediction was modified if a homologous protein in the TOPDB database had an annotated signal peptide that was not predicted by SignalP, or *vice versa*, if the corresponding TOPDB entry had not contained a signal peptide, but it was predicted by SignalP. In the next step, the TMPs were filtered by the consensus filter method described in *Methods*. If the filter method resulted in TMP, CCTOP was used to create the final topology of the TMP.

The HTP database is stored in the XML format. Every entry contains not only the final prediction results, but also the cross-references, the prediction results of the ten selected methods, and the alignment used to mirror the experimental topology data into the human sequence. The HTP’s XSD Schema definition can be downloaded from the HTP home page as well.

### Comparing the human transmembrane proteome database with other resources

Three resources were incorporated into the comparison: i) proteins in the UniRef 90 Human Proteome which have 2 or more transmembrane helices predicted by the TMHMM method [53] (TMHMM set); ii) data from a recently published human membrane protein analysis system, called HMPAS [54] (HMPAS set); iii) human membrane proteins, which have membrane subcellular localization indicated in the UniProt database (UniProt set). For the comparison and for creating a Venn diagram we used the Venny program [55] available at the URL http://bioinfogp.cnb.csic.es/tools/venny/index.html.

### Website of the human transmembrane proteome

The website of the HTP database was written in C++, using the Wt webtoolkit C++ programming library [56] and the in-house made XBuilder library. Recently we have created two complex web applications for investigating protein 3D structures and residue-residue interactions [57] and for the PDBTM database [1], where both program libraries have been successfully utilized for serving a graphical user interface through the web. The C++ source code of the CCTOP prediction method can be downloaded from the home page of the HTP database.

## Results and discussion

### Benchmark sets

Most of the topography and topology prediction methods developed so far have been trained and/or tested on small benchmark sets, mostly on the so-called TMHMM 160 protein set [29], which contains three types of data: entries originated from the Möller data set; a prokaryote data set; and other individually collected proteins. Here, we established a new benchmark set, comprising sequences and topologies of human TMPs only by searching sequences homologous to the human sequences in the TOPDB database. The resulting TMP set was divided into a “structure” and an “experimental” benchmark set. The former part contains human TMP sequences, whose homologues in the TOPDB database have a 3D structure, while the experiment benchmark set contains human proteins whose homologous partner in the TOPDB database has experimentally verified topological data only. In order to guarantee the same topology for the human protein and the protein in the TOPDB database the parameters of the BLAST search were set very strictly. In the case of the structure benchmark set, homologous proteins from the TOPDB database were used, of which all TMHs were defined in the corresponding PDB file.

These two benchmark sets were merged and then expanded with globular proteins from the PDBSelect dataset [40] to prepare a benchmark set for filtering purpose. This “filtering benchmark set” models the human proteome in the sense that it contains TMPs and globular proteins in the same ratio as was shown earlier by other studies (25 % TMP and 75 % non-TMP).

The topology and the sequences of these three benchmark sets can be downloaded from the website of the HTP database.

### The constrained consensus topology prediction method

The Constrained Consensus Topology prediction method (referred as CCTOP hereinafter) is composed of three basic steps. The first step is the prediction of signal peptides. Depending on the signal peptide prediction’s output, in the case of a positive result the signal peptide is cut before any further investigation, because most of the topology prediction methods confuse signal peptides and TMHs. Next, CCTOP makes a decision as to whether the investigated protein sequence encodes a TMP or a non-TMP. The final step is the topology prediction. CCTOP, as its name shows, utilizes several methods to perform these tasks and incorporates the results of already known topological data or bioinformatical evidences into the final topology prediction as constraints. We describe the details of these steps and the results of these predictions in the following sections.

### Signal peptide prediction

We have tested several prediction methods on the merged human benchmark sets (structure and experiment). Because the prediction accuracies of these methods are high, and because we cannot increase the accuracy by combining them, CCTOP utilizes SignalP prediction with a single modification. That is, if there is a protein homologous to the investigated one in the TOPDB database then the signal peptide data from TOPDB are used instead of SignalP results. Table 1 contains the results of the various predictions.

**Table 1:** Results of the various signal peptide prediction methods on the human benchmark set

	Philius	Phobius	SignalP	SPOctopus
**TP**	204	196	194	168
**FP**	24	21	14	22
**TN**	234	237	244	235
**FN**	12	20	22	49
**Sensitivity**	0.94	0.91	0.90	0.77
**Specificity**	0.91	0.92	0.95	0.91
**MCC**	0.84	0.83	0.85	0.70

TP: number of true positives; FP: number of false positives; TN: number of true negatives; FN: number of false negatives; specificity is TN/(TN + FP); sensitivity is TP/(TP + FN); MCC: Matthew’s Correlation Coeffitient is (TP*TN-FP*FN)/sqrt ((TP + FP)*(TP + FN)*(TN + FP)*(TN + FN))

### Discrimitaning between transmembrane and non-transmembrane proteins

Transmembrane topology prediction methods are commonly used to discrimitaning between TMPs and non-TMPs. However, as we pointed out earlier, [18] it isn’t a good practice, since most of the methods are trained only on TMPs and not on mixed sets of TMP and non-TMP sequences. Here we tested several methods on the filtering benchmark set described in the previous section and the best ones were combined in order to reach an even better accuracy. As it can be seen in Table 2, the specificity of each method tested is high, while their sensitivities and Mathews correlations are somewhat lower. However, we achieved higher accuracy by combining the tested methods, and reached 99 % both for sensitivity and specificity, respectively (Table 2, Column CCTOP). PRODIV and HMMTOP were not tested, as they are not able to distinguish transmembrane and globular proteins, because the key hypothesis in these algorithms is that the investigated protein is a TMP.

**Table 2:** Results of the various prediction methods for filtering TMPs on “filtering benchmark set”

	MEMSAT-SVM	Octopus	Philius	Phobius	PRO	Scampi-single	Scampi-multi	TMHMM	CCTOP
**TP**	469	455	460	462	417	469	454	451	467
**FP**	51	40	24	20	28	26	40	21	21
**TN**	1371	1382	1398	1402	1394	1396	1382	1401	1401
**FN**	5	19	14	12	57	5	20	23	7
**Sensitivity**	0.99	0.96	0.97	0.97	0.88	0.99	0.96	0.95	0.99
**Specificity**	0.96	0.97	0.98	0.99	0.98	0.98	0.97	0.99	0.99
**MCC**	0.93	0.92	0.96	0.96	0.88	0.96	0.92	0.94	0.96

See legend of Table 1

### Topology prediction

We have also tested the topography and topology prediction accuracies of the various methods available on the internet either online or in locally executable form. Although the prediction accuracies of most of these methods were reported above 85–90 %, in the human benchmark sets their accuracies are somewhat lower (see Table 3 and Table 4). This could be the result of the fact that these prediction methods were trained on sets containing mostly prokaryote sequences. For comparison, we have tested another consensus based prediction methods, TOPCONS, as well. The developed CCTOP prediction method enhances the accuracy by utilizing two phenomena. As it was shown by several publications, consensus approaches usually work better than simple methods by eliminating the sporadic errors of individual methods. We checked the effect of the number of the selected topology methods on the final accuracy of the consensus method, and find that the accuracy is saturated with the number of the used methods (Additional file 3). Therefore, CCTOP is based on the prediction results of ten topography and/or topology prediction methods. The novelty of CCTOP is that it incorporates these results as constraints in a probabilistic framework provided by hidden Markov models. Another way for enhancing prediction accuracies is the integration of the already available topological data and other bioinformatical evidences into the prediction as constraints in the same probabilistic framework. As it is shown in Table 4, by using these data the topology prediction accuracy raises by more than 15 % on the experimental data set. By using the reliability value calculated by the CCTOP algorithm, the reliability of the individual predictions can be measured as well, and we are able to select the most accurately predicted subset of the human transmembrane proteome (see Reliability of the HTP database). This feature enhances the usability of the CCTOP even more.

**Table 3:** Topology prediction results on the structure benchmark set

	HMMTOP^a^	Membrain	MEMSAT-SVM	Octopus	Philius	Phobius	Pro	Prodiv	Scampi-Msa	TMHMM	TOPCONS	CCTOP^a^
**Sens/res**	0.97	0.99	0.96	0.97	0.96	0.97	0.9	0.97	0.99	0.94	0.97	0.99
**Spec/res**	0.97	0.94	0.99	0.98	0.98	0.97	0.98	0.97	0.98	0.99	0.98	0.99
**MCC/res**	0.97	0.96	0.97	0.97	0.97	0.97	0.94	0.97	0.98	0.96	0.96	0.99
**Acc**_**Tpg**_ **/prot**	84	76	80	83	81	79	66	82	89	76	78	93
**Acc**_**Top**_ **/prot**	81	0	68	82	74	77	51	67	88	70	76	92

Prediction accuracies of the various topology prediction methods on the structure benchmark set. Sens/res, Spec/res, and MCC/res mean per-residue sensitivity, specificity, and Matthew correlation coefficient, respectively. Acc_Tpg_/prot and Acc_Top_/prot mean per/-protein topography and topology accuracies multiplied 100, respectively (^a^predictions were made without topological constraints)

**Table 4:** Topology prediction results on the experimental benchmark set

	HMMTOP^a^	Membrain	MEMSAT-SVM	Octopus	Philius	Phobius	Pro	Prodiv	Scampi-Msa	TMHMM	TOPCONS	CCTOP^a^	CCTOP
**Sens/res**	0.92	0.88	0.95	0.92	0.92	0.92	0.9	0.83	0.94	0.88	0.93	0.96	0.96
**Spec/res**	0.92	0.97	0.92	0.97	0.96	0.94	0.96	0.94	0.91	0.97	0.94	0.96	0.97
**MCC/res**	0.92	0.92	0.94	0.94	0.94	0.93	0.93	0.83	0.92	0.92	0.91	0.96	0.96
**Acc**_**Tpg**_ **/prot**	66	67	70	73	72	68	67	57	64	67	64	82	85
**Acc**_**Top**_ **/prot**	57	0	59	63	68	64	52	44	60	60	59	80	84

Prediction accuracies of the various topology prediction methods on the experimental benchmark set. Sens/res, Spec/res, and MCC/res mean per-residue sensitivity, specificity, and Matthew correlation coefficient, respectively. Acc_Tpg_/prot and Acc_Top_/prot mean per/-protein topography and topology accuracies multiplied 100, respectively (^a^predictions were made without topological constraints)

### The human transmembrane proteome

Using CCTOP as an accurate and precise filtering, signal peptide and topology prediction method on human sequences, we investigated all human sequences in the human proteome defined by the UniProt database (version UniRef90 2013, March). We filtered 4998 sequences as TMPs, which is 26 % of the human proteome as expected from earlier studies.

**Fig. 1:**
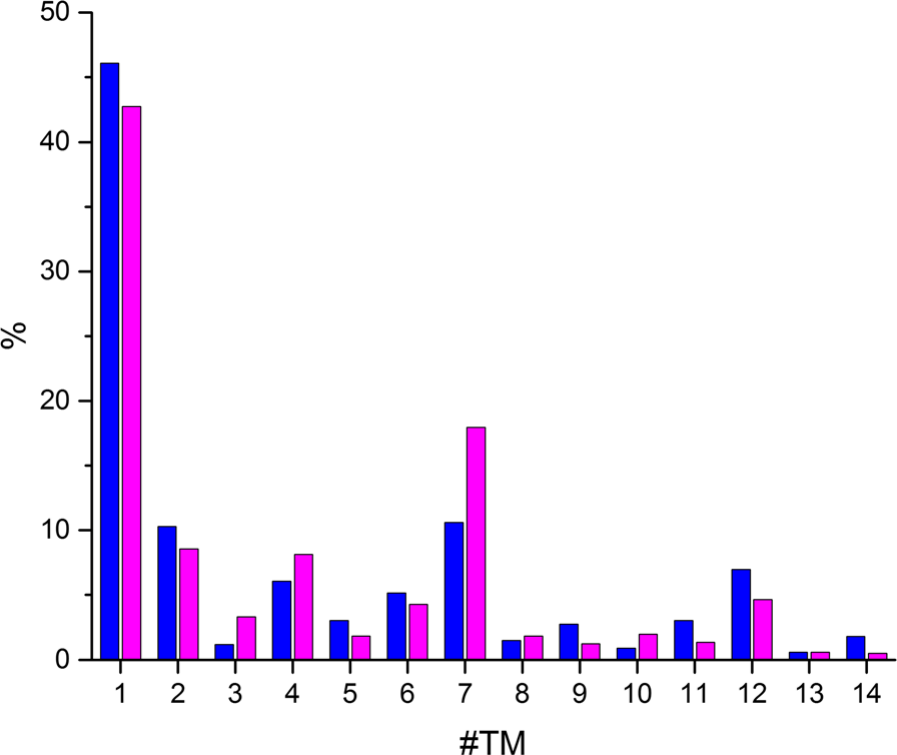
Distribution of the number of transmembrane helices. Distribution of the number of TMHs in TMPs in the experimental benchmark set (*blue*) and the predicted human transmembrane proteome (magenta)

The distributions of the number of TMHs in the experimental set and in the predicted human proteome are very similar (Fig. 1). Notably, results on the benchmark sets are similar to the results on the whole human transmembrane proteome. The most prevalent class is the one TMH containing proteins. According to the GO annotation, proteins in this class are involved in cell adhesion, in biosynthetic and metabolic processes or function as receptors. According to the WEB-based GEne SeT AnaLysis Toolkit, the most enriched diseases related to this class of TMPs are various immune system diseases, virus diseases, infections, necrosis and transplantations [58]. The second most abundant class is the seven TMH class, which contains the largest TMP family, namely the GPCR protein family. The higher values of the even numbered TMH classes above ten indicate that the genes of these proteins should be the results of tandem duplication. These results are consistent with previous studies [28, 47], but slightly differ in the number of 7 TMHs containing proteins. This may be the results that MEMSAT-SVM has the lowest prediction accuracy on those protein in the structure benchmark set which contains 7 TMHs.

**Fig. 2:**
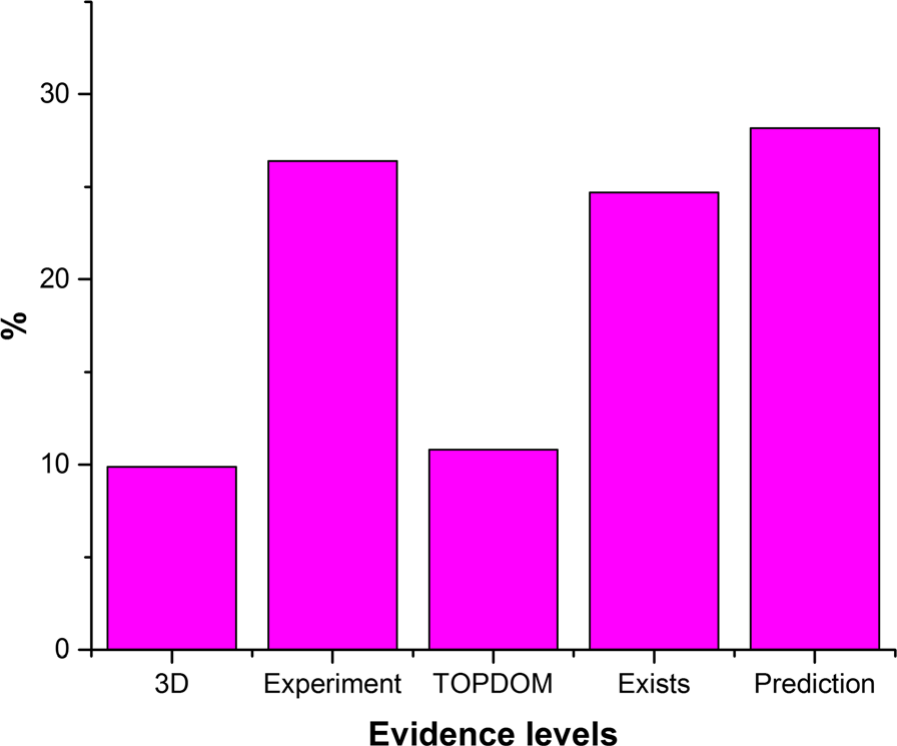
Distribution of evidence levels. Distribution of evidence levels in the predicted human transmembrane proteome

In the HTP database, predictions were categorized into five evidence levels according to the type of the used topology data or the lack of this information. These evidence levels are 3D, Experiment, TOPDOM, Exists and Prediction. The most certain predictions are on the 3D level, where 3D structure of the given human TMP has already been determined, or a 3D structure of a homologous protein has already been solved. In the latter case, strict parameters are used to generate an alignment and to mirror the topology information from the known sequence into the unknown human TMP, and the topology data of the homologous protein in the TOPDB database has been used as constraint in the final prediction. The next evidence level is the Experiment level, when the 3D structures of the protein itself or of homologous proteins are not known, but some molecular biology experiments were made. Entries are marked with TOPDOM evidence level, if bioinformatic evidences can be found that can be used as a strong argument to define the topology of the full protein or some parts of it. The experimental topology data is collected in TOPDB, while bioinformatical evidence can be generated using the TOPDOM database and its search engine. The Exist evidence level is used when constraints do not exist at all in the TOPDB or in the TOPDOM databases, but there is some evidence that the protein exists. The last evidence level is the Prediction level. In this case, there is no evidence of the existence of the protein; therefore, both the amino acid sequence and the topology are predicted from the human genome sequence. According to the distribution of the evidence level in the HTP database (Fig. 2), almost half of the protein topologies belong to the 3D or Experiment levels, which are the most reliable parts of the database.

### Reliability of the HTP database

**Fig. 3:**
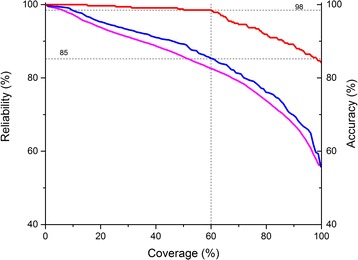
Correlation between accuracy and reliability. Predictions are sorted by descending reliability order. Then the topology accuracy were calculated for each subset containing predictions from the most reliable to the least one. The x-axis measures the relative size of the subset to the whole size of subset or of the human transmembrane proteome, the y-axes measure the topology accuracy measured on the subset and the least reliability value in the same subset. *Red* and *blue line* are the topology accuracies and smallest reliabilities measured on benchmark sets, respectively. Magenta line is the smallest reliability measured in the subset of human proteome. The vertical dashed line is at 60 % coverage and its cross with the topology accuracy curve (*red line*) at 98 % and with the reliability curve at 85 % are indicated with horizontal dashed lines

Besides the utilization of consensus prediction methods and topology data as constraints, a unique feature of the CCTOP algorithm is the calculation of the reliability of the prediction. It is done by summing up the posterior probabilities through the path of states of the final prediction, determined by the Viterbi algorithm. By sorting the predictions according to the calculated reliabilities, the prediction accuracies on the most reliable subsets are decreasing monotonously (Fig. 3, red). According to this result, CCTOP can predict the topology with accuracy above 98 % for more than 60 % of the benchmark set, and using the reliability values, we can identify these most accurately predicted proteins, without knowing the topology. The reliability-coverage curve shows similar shape on the benchmark set and the whole human transmembrane proteome (Fig. 3, blue and magenta), therefore it is plausible that the predicted topologies in HTP database may be as accurate as in the benchmark set, i.e. more than 60 % of the predicted topologies’ accuracies may be over 98 %. Those entries, whose reliabilities are above 85 % belong to this highly accurate predicted subclass of the human transmembrane proteome (see Fig. 3, dashed lines).

### Comparing the HTP database with other resources

**Fig. 4:**
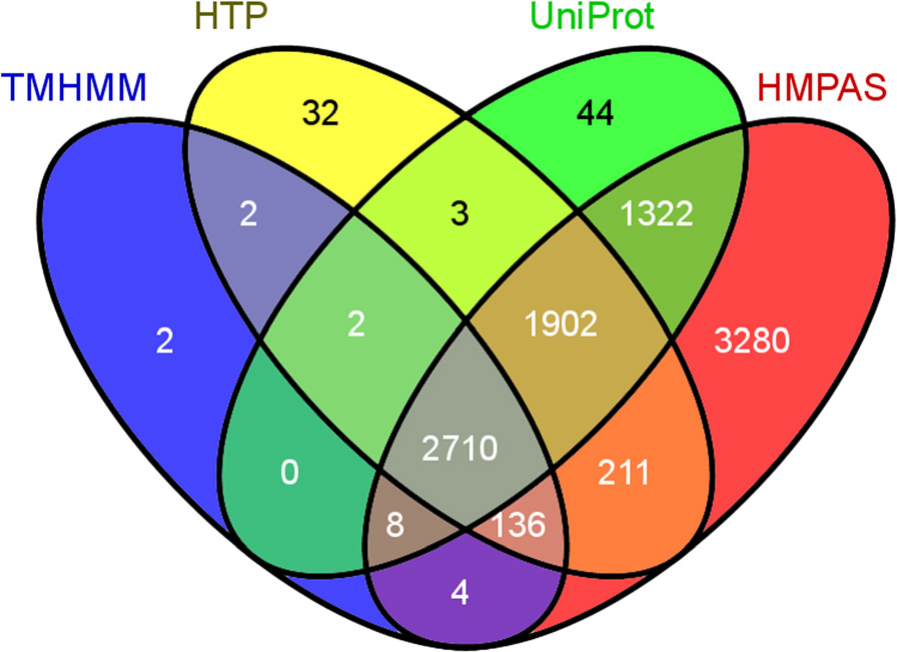
Comparison of different predictions of the human transmembrane proteome. Venn diagram of the various predicted human transmembrane proteomes

We have compared three other human transmembrane proteomes published so far with the data in our HTP database (Fig. 4). For structural genomics of human α-helical transmembrane proteome the Sali lab used a simple algorithm to create an initial set for their purpose [52]. As it can be seen in Fig. 4, there are only 14 proteins that are in this TMHMM set, but were not identified by CCTOP algorithm as TMP. However, the TMHMM set missed 2148 TMPs, most of which have only one transmembrane region. As the HMPAS data set contains 4500 more proteins than HTP, because in this database every protein is listed that has any association with the membrane, i.e. contains the “membrane” GO annotation. In the UniProt database there are 1342 entries containing “Subcellular location: membrane” annotation, which we did not identify as TMP. However, only 28 % of these entries have additional annotation in the feature table (FT line), localizing the transmembrane region in the sequence, and only three of them are based on experimental results, the others are based on predictions and/or similarities. The transmembrane proteome published by Faberger et al. is not presented on Fig. 4, because none of the downloadable files contained the type of the proteins they characterized, nor the topologies of TMPs.

### The website of the human transmembrane proteome database

**Fig. 5:**
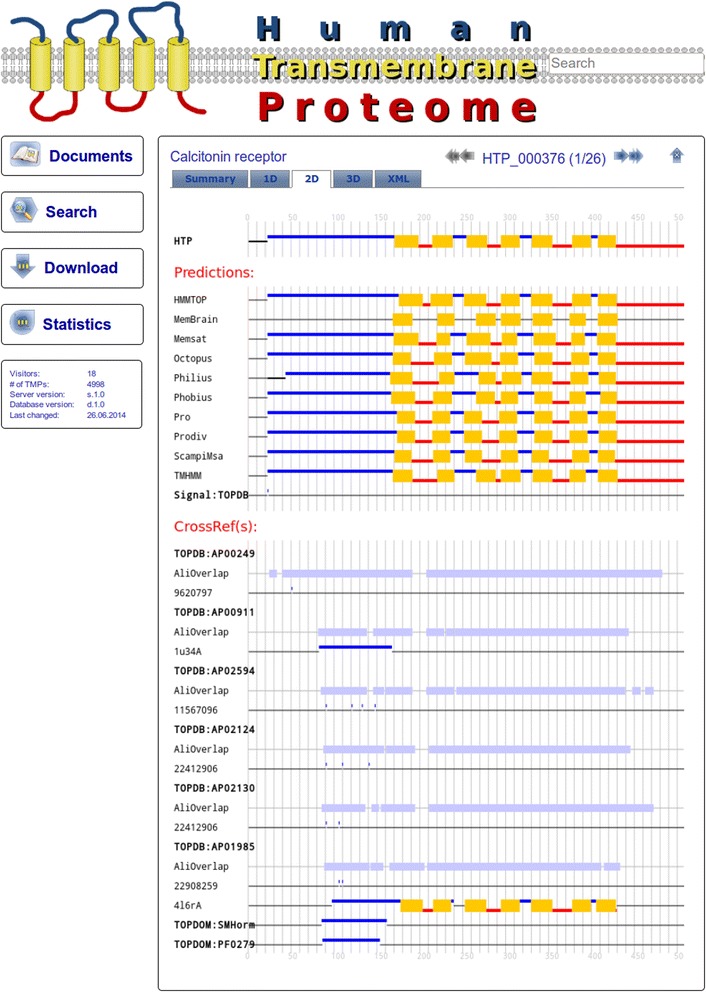
Website of the Human Transmembrane Proteome. An example screenshot from the HTP home page. *Yellow rectangles*, *blue* and *red lines* represent TMHs, extra-cytosolic and cytosolic regions, respectively

The homepage of HTP database is available at the URL: http://htp.enzim.hu. Besides the option to download the raw data, we have created an interactive graphical user interface (GUI) for the visualization of the collected topology data as well as the results of the various prediction methods for each protein. This information is shown on a 3D or 2D graphical interface or as raw xml files. All data are searchable in simple mode or in advanced mode and the search results can be visualized separately, or downloaded as one archive file. All functionalities of the HTP website are described in detail in its user manual. A representative screenshot of the HTP website can be seen on Fig. 5.

### Future directions

We would like to update HTP database regularly, following the three source databases’ (TOPDB, TOPDOM, UniProt) update. During the preparation of HTP database UniProt had released a new human proteome, containing the alternative splice variants of genes. We would like to incorporate these splice variants into the HTP database as well.

## Conclusions

The CCTOP algorithm is a novel method for predicting transmembrane protein topology. Besides utilizing 10 different state-of-arts methods, experimental and bioinformatic information is incorporated into the prediction from PDBTM, TOPDB and TOPDOM databases. The CCTOP algorithm was tuned and benchmarked on newly compiled human protein sets and was shown to have the highest accuracy among other tested state-of-art and consensus methods.

Using the CCTOP algorithm on the human proteome, it predicted that 4998 (26 %) proteins contain TMH(s). The gathered information was used to construct the HTP database, which is available at the URL: http://htp.enzim.hu. It contains all human α-helical transmembrane proteins, with established topology by the CCTOP algorithm. In addition to download the raw data, a graphical user interface was created for the visualization of the collected information. Various search and browse modes have been added in order to simplify the gathering of the desired information. We are planning to update the database regularly, following the updates of UniProt, as well as to extend its content.

## Reviewers’ comments

### Reviewer comment 1: Dr. Sandor Pongor

**Report form:** In this manuscript Dobson et al. present a new method for predicting the topologies of transmembrane proteins and the application of this approach on the human proteome. Transmembrane proteins play important roles in human body and are the target of half of the drugs currently available on the market; meanwhile there are only hundred TMP structures solved. Therefore, the computational approaches and curated databases, like the one presented in this manuscript, are highly needed. According to the authors, there method has the highest accuracy among the dozen or so currently available programs designed for the purpose. However, some issues need to be addressed:

**Authors’ response:** We thank reviewer for this comment.

**R1:** Is the use of CCTOP for filtering of membrane proteins significantly better than individual methods? The authors need to describe how methods were chosen for evaluation their consensus method.

***Authors’ response:****We have prepared Additional File**3**by combining the results in a Venn diagram of methods with Matthews correlation coefficients above 0.93. In Additional File**3**, the results of all triplet combinations of the selected four methods can be found. As it expected, and can be seen from these data, combining the various algorithms decreases the false negative (FN) and false positive (FP) ratios. However, the true positive (TP) ratio decreases as well. Therefore, there should be an optimal number of the combined methods. Using a simple majority decision algorithm for three methods out of the selected four ones, the highest accuracy could be reached if the three methods were TMHMM, Scampi and Phobius.*

*Regarding the significance of our filtering algorithm, we note that our aim was to predict the human transmembrane proteome as accurate as it could be. Since the size of the human proteome is about 20,000, one percent difference between the prediction accuracies of two methods would result two hundred incorrectly predicted proteins.*

**R1:** Combined signal peptide and topology prediction methods show better performance than applying them individually. However, CCTOP algorithm separates these two predictions. It would have resulted in better performance, if CCTOP had used a hidden Markov architecture similar to Phobius or SPOctopus.

***Authors’ response:****In the human benchmark set the performance of these two methods are lower, than of Philius and SignalP4.0 (see Table**1**) regarding the signal peptide prediction accuracies. However, CCTOP algorithm similarly to Phobius and SPOctopus, exploits the result of the signal peptide prediction by utilizing an extra-cytosolic constraint at the N-terminus of the sequence. We have to choose this solution, because CCTOP and the other HMM based prediction methods apply different learning schema (unsupervised vs supervised, respectively), therefore we cannot use a HMM architecture developed for supervised learning.*

**R1**: Are the prediction performances of the applied single methods reliable or do they only show the similarity between the training set used during their development and the benchmark sets used in this manuscript?

***Authors’ response:****Since we could not retrain the individual methods on our benchmark sets, we can answer this question only indirectly. We have prepared a new set from the Structure benchmark set by removing those proteins, which have 40 % or higher sequence identity with any sequence of the training sets used by the 10 prediction methods. Altogether 61 proteins were removed. Then we tested the prediction accuracies of the methods on this smaller set. To estimate the effect of the smaller set, we prepared hundred random set, by removing 61 proteins randomly from the Structure benchmark set, and tested the accuracy on these random sets as well. The following diagrams show the results:*





*As it can be seen, we did not get significantly better or worse topology prediction accuracy by removing the 61 protein used for training the various methods, than in random cases, indicating that the used methods are not overfitted to the structure benchmark set.*

**R1**: The used topology prediction methods are apparently not cross-validated on the current experimental and structure benchmark set. In this case, how can the reliability of the performance of a consensus predictor be estimated?

***Authors’ response:****See our answer to the previous question. Moreover, the fraction of those TMPs in the experimental benchmark set, which were used formerly in training, is smaller, and removing these proteins does not affect the prediction results at all. As the weights for the various methods used for the final CCTOP prediction were calculated from the topology prediction results on structure benchmark set, the experimental benchmark set can be regarded as an independent test set.*

**Quality of written English**: Acceptable

**Quality of Figures**: Acceptable.

*Reviewer comment 2: Dr. Michael Galperin*

**Report form:** The manuscript by Dobson and colleagues describes the newly created Human Transmembrane Proteome (HTP) database (http://htp.enzim.hu), a potentially useful resource. However, the current versions of the database and of its description contain serious flaws and would be misleading for the readers and the potential users. The database and the accompanying manuscript must be carefully revised before this work could be considered acceptable for publication in Biology Direct.

I have checked the performance of the database using the well-known family of G-protein coupled receptors (GPCRs) whose members contain 7 transmembrane (TM) segments. To my great surprise, the HTP reported 5 TMs for GP148_HUMAN and 8 TMs for EDNRB_HUMAN (in both cases UniProt predicts 7 TMs). A quick look at the underlying data showed that for GP148_HUMAN, five TM prediction methods used by HTP found 5 TMs, whereas other five predicted the same 5 TMs plus two more for a (correct) total of seven. In the case of EDNRB_HUMAN, again five methods correctly predicted 7 TMs, and the other five added an additional TM at the N-terminus, a likely signaling peptide. The consensus method (CCTOP), developed by the authors, weighted results of 5 methods against the results of other 5 methods and, in each case, made the wrong choice. This result not only questions the quality of the CCTOP tool, it also shows that a naive user who would trust the HTP output would end up with a worse prediction than the one available in UniProt.

***Authors’ response:****We thank the reviewer for pointing out this flaw in the HTP database. After careful investigation of the CCTOP results for these and other 7TMHs proteins, we found a bug in our code, which caused this type of misprediction. After eliminating this bug, we recalculated the CCTOP’s topology prediction for GPCRs and other affected proteins. Altogether 76 predictions were modified. We updated Fig.**1**as well. Moreover, we prepared a GPCR test set from UniProt database by selecting those human proteins that are marked as “reviewed” in the UniProt file, contain the “G protein coupled receptor” words in the file and featured in HTP database (870 proteins) then checked how many cases can be found 7TMH in the UniProt files themselves and in the prediction results of the various methods:*

**Table:** 

**Method**	**Acc(%)**
SwissProt	90.23
HTP	89.89
ScampiMsa	83.45
PRODIV	82.30
Philius	77.93
MemBrain	70.57
TMHMM	70.46
PRO	69.77
Phobius	61.38
Memsat	49.20
Octopus	45.75
HMMTOP	41.61

*As it can be seen, now HTP database is as accurate as Uniprot, regarding GPCR proteins.*

**R2:** Therefore, I would strongly suggest showing, instead of a single ‘# of TM segments’ in the Summary view, all the possible numbers of TMs predicted by various tools. In addition, the 2D view should be made the default one. In the current display of Search results, it is not even explained that the user should click inside the box to see the choice of 1D, 2D or 3D view.

***Authors’ response:****There is no default tab in the server for the entry view, the last used tab is shown if a new protein is selected from the search list or entered directly by url. Therefore, if a user thinks that the 2D tab is more useful than the Summary tab, the 2D tab simply can be used as default. Despite of it is explained in the server manual page (**http://htp.enzim.hu/?_=/documents/sman/sman_listviewer.html**) at point D that “User can click to the icon in order to open the Entry Viewer Panel.”, we made this step more evident by putting a clickable arrow behind the icon and make the headline of each search result clickable as well.*

**R2**: Obviously, the above examples suggest that the quality measures described in the manuscript are likely to be biased. However, I would argue that these measures do not belong into this manuscript in the first place.

***Authors’ response:****As we described above, there was a bug in the source of the CCTOP prediction method causing this type of error. However, we would argue that the reliability values calculated for each entry are worthy, since the reliability values of both entries pointed by the Referee were low, which call the attention of the potential user, that the prediction is not certain. Since the HTP database contains the prediction results of CCTOP algorithm, it is evident that the prediction can make mistakes. As we shown in Fig.**3**, the prediction accuracy for the 60 % of the entries are above 98 %, which reliability value is above 86 %. For the two examples pointed by the Referee the reliability values were below this limit, showing the usability of the reliability value. Moreover, since the CCTOP algorithm uses TOPDB and TOPDOM databases, the updating of these two databases may cause a change of CCTOP prediction on human proteins as well. Therefore, we are planning to rerun CCTOP algorithm from time to time after updating TOPDB, TOPDOM or the UniProt proteome source.*

**R2:** Basically, the reviewed manuscript combines two distinct parts that do not fit together very well. One part is the description of the tools for TM segment and topology prediction that have been used in the HTP database. I have no major complaints about this part, although it would be useful to carefully examine all the cases where different tools produce different results and identify any potential sources of systematic error.

***Authors’ response:****Reliability values correlate well for cases when the different prediction methods produce different results, and we could not detect any systematic error of them.*

**R2:** In my opinion, Table S1 makes much more sense than any tables included in the main text (although one could question whether SOSUI is really inferior to other tools).

***Authors’ response:****As it is explained in Table S1, the SOSUI server was instable during the development of CCTOP algorithm; it froze several times and was unavailable for days or even weeks. This was the reason that we finally omitted it from the CCTOP algorithm.*

**R2:** As an example, the authors do not specify which version of SignalP they have used. In fact, SignalP version 4 has been specifically modified as compared to version 3 to allow better discrimination between cleavable signal peptides and uncleavable ones that stay in the protein and form N-terminal TM segments. Our own recent analysis showed that this change resulted in a substantial improvement of signal peptide prediction by version 4, as judged by proteogenomics-based identification of signal peptides [Ivankov et al., Environ. Microbiol. 2013, 15 (4):983–990, PubMed ID: 23556536]. The second part is a purely bioinformatics exercise that includes benchmarking of various programs and is supposed to show the superior performance of the CCTOP tool. As explained above, this section looks suspicious and the CCTOP tool does not seem to add much value.

***Authors’ response:****We used SignalP version 4.1 in the CCTOP algorithm, but indeed, this information was only indirectly presented in the manuscript by citing the paper describing the SignalP 4.0 version. Now we added this info to the manuscript.*

**R2**: In summary, the HTP database can be made into a useful resource. To accomplish that it would need to be displaying all prediction results generated by various tools instead of trying to arbitrarily select one result over the other. The description of such a database would be a welcome contribution, worthy of publication in Biology Direct. In contrast, the description of the CCTOP tool and its benchmarking should be made into a separate paper that would be more suitable for a specialized bioinformatics journal.

***Authors’ response:****We are planning to describe the details of the CCTOP algorithm separately, but currently this is not yet available. We think, the short description of the algorithm in the present manuscript help understanding how the database was created.*

**Quality of written English**: Acceptable

**Quality of Figures**: Acceptable.

*Reviewer comment 3: Dr. Pascale Gaudet (nominated by Dr Michael Galperin)*

**Report form**: I have reviewed the paper by Dobson, Reményi and Tusnády entitled “The Human Transmembrane Proteome”. The paper describes a new database of human transmembrane proteins. As described by the authors, transmembrane domains are difficult to assess experimentally, so accurate methods for annotation of transmembrane proteins based on experimental data and predictions are very valuable.

**Comments on the manuscript:**

Please clarify the following points:

**R3**: In the Results section of the Abstract: authors quantify the accuracy of their method using ‘filtering’ and ‘per protein topology prediction’ measures. Although this is described in the main section of the paper, these terms should be defined briefly, or removed. Also, it is not clear to me whether the next sentence (“Besides predicting topology, reliability of the predictions is estimated as well, and it is demonstrated that the accuracies of more than 60 % of the predictions are over 98 % on the benchmark sets”) refers to the same measures. Please clarify.

***Authors’ response:****We change ‘filtering’ to ‘discriminating between transmembrane and non-transmembrane proteins’. The ‘per protein topology prediction’ category is commonly used in the field of transmembrane topology prediction (see for example pmid:15215532). We modified the next sentence to clarify its meaning.*

**R3**: In the Background section: It is not clear what study this sentence refers to “Although the reported per protein transmembrane topology prediction accuracies of the various algorithms were shown to be above 80 %, they reached rather low prediction accuracies on a human benchmark set (see below).”; please be more specific with respect to the source of this data.

***Authors’ response:****We meant the various state-of-the-art prediction methods used by the CCTOP algorithm and listed in Additional file**1**. We have put in link to Additional file**1**in this sentence in order to clarify this issue. The table in the Additional File 1 contains the references to the reported high prediction accuracies.*

**R3**: On p. 6, when describing the creation of the database, the authors mention a “consensus method”. This consensus method must be defined.

***Authors’ response:****We mean CCTOP here. We changed the sentence to make this point clearer.*

**R3**: On p. 6, authors also mention a newly established benchmark of more than 450 human proteins. How was this benchmark defined? It is available for other researchers to test their prediction algorithms?

***Authors’ response:****It is described in the “Methods, Preparation of the benchmark data sets” section of the manuscript. The benchmark sets can be downloaded from the website of the HTP database.*

**R3**: Methods: On page 7, the authors mention that they used UniRef90 Human Proteome from March 2013. Are the authors planning to re-run the analysis on a more up-to-date version of the database?

***Authors’ response:****Yes, we are planning to update the database regularly, as well as to extend its content. We add this information to the end of “Conclusion”.*

**R3**: On p. 7, the authors describe the use of PDBTM, TOPDB and TOPDOM data. Please cite which version was used for each of these databases.

***Authors’ response:****We added the appropriate version numbers into the manuscript.*

**R3**: On p. 9, authors mention that the consensus algorithm was chosen by testing “dozens of combinations of these approaches”. The actual consensus algorithm should be described. It seems like the section “ Constrained Consensus Topology prediction” describes the algorithm; it may be that adding a transition sentence would clarify this point.

***Authors’ response:****We have prepared a new document (AdditionalFile_3.doc), which describe the algorithm of the discrimination between transmembrane and non-transmembrane proteins, and linked this file to the sentence cited by the Referee in the manuscript.*

**R3**: p. 12, authors describe using Uniprot annotations to detect (and cleave) signal peptides, and “then the signal peptides have been predicted by SignalP”. Is the Signal P prediction run *after* the data is processed from UniProt ? Does that provide any additional signal peptides? That would be surprising (unless different cut-offs are used), since UniProt also uses SignalP to predict signal peptides.

***Authors’ response:****In the manuscript we wrote that “All transit peptides have been cleaved using UniProt annotations, then the signal peptides have been predicted by SignalP”. That is, we use UniProt only for removing transit peptides and not for identifying signal peptides. For signal peptide detection we use the SignalP method and the information from TOPDB database. This is now made clearer in the manuscript.*

**R3**: In that same section, that authors describe modifying the prediction if an annotated signal was not predicted by SignalP; in UniProt there may be signal sequences that have been shown experimentally, yet not predicted by SignalP; I expect that extra step may create both false positives and false negatives annotations.

***Authors’ response:****We use the information from the TOPDB database to modify the result of SignalP, and not from the UniProt, moreover the order is the opposite: first we generate the SignalP prediction and this prediction is modified, if the TOPDB database contain contradictory data. In this way FP and FN are lower than simple use of SignalP prediction.*

**R3**: p. 14: Please provide a reference for the TMHMM160 protein set.

***Authors’ response:****This set was compiled for the TMHMM algorithm, and is described in J Mol Biol 2001, 305:567–80. We inserted this reference.*

**R3**: p. 18: It is misleading that the ‘Experiment level’ evidence level contains both experimental data as well as bioinformatics evidence. Is it possible to distinguish between these two categories?

***Authors’ response:****We thank the Referee for this suggestion. We introduced a new evidence level, called TOPDOM for entries which contain cross reference(s) only to the TOPDOM database and updated the text and Fig.**2**in the manuscript accordingly.*

**R3**: Tables 1 and 2 are missing a legend.

***Authors’ response:****We insert the missing legends.*

**R3**: Fig. 3 Title: Remove the two instances of “the”.

***Authors’ response:****We have amended the title of Fig.**3**.*

**R3**: Fig. 3 legend is not clear: what is the difference between the blue line and the red line? Please rephrase.

***Authors’ response:****We have rephrased the legend of Fig.**3**.*

**R3**: Fig. 3: How is the reliability on the entire human proteome evaluated?

***Authors’ response:****We did not evaluate the reliability on the entire human proteome. Reliability was determined in each entry in the HTP database, and then entries were sorted according to their reliability values in descending order.*

**Grammatical and typological corrections**

**R3**: Generally: The term ‘extracellular’ is more commonly used than ‘extracytoplasmic’ for protein segments outside the cell. This is also the UniProt nomenclature, see http://www.uniprot.org/help/topo_dom.

***Authors’ response:****We use extracytoplasmic not just for protein segment outside the cell, but for every protein segment that is the opposite site than the cytosol,* i.e. *the inside of the various somes (endosomes, lysosomes, microsomes etc.), intermembrane space of mitochondrium, cysternal space of endoplasmic/sarcoplasmic reticulum. For details, see the various membrane types in the documentation on the website of TOPDB database (**http://topdb.enzim.hu/?m=docs&mm=membranes**).*

**R3**: − Generally: The authors seem to use ‘homepage’ to mean ‘website’.

***Authors’ response:****We have replaced ‘homepage’ by ‘website’ through the manuscript.*

**R3**:

- p. 3: Replace “should” by “must” or “may” in the sentence “This should be because the topology prediction (…)”
- p. 3: Remove comma in the sentence “Signal peptides control proper targeting of proteins, which are destined toward the secretory pathway”.
- p. 4: Delete “may” in the sentence “These modifications may occur only at the extra-cytosolic side of proteins”
- p. 4: Delete “the” in the phrase “experimentally established topological data into the prediction methods”
- p. 5: Period missing after “a fully automated algorithm”.
- p. 5: Delete “which” in the phrase “domains in the SMART database which were found in soluble proteins”
- p. 5: Replace “kind” by “type” in the phrase “kind relevant to membrane protein topology”
- p. 5: What is meant by “classed material” in the phrase “unavailability of classed material.” ? Please rephrase.
- p. 5: The meaning of the sentence “Their results showed that the five prediction methods agreed more on the 6 benchmark set than the various genomes.” is not clear; please rephrase.
- p. 6: Delete “used” in the phrase “how similar is the data set (e.g. a genome) to the used benchmark set”
- p. 6: Replace “They” by “The authors” in the phrase “They found that the available test set”
- p. 6: Replace “of” by “on” in the phrase “information of human ?-helical TMPs”
- p. 6: Replace “sequential” by “sequence” in the phrase “glycosylation sequential data”
- p. 8, Remove comma between “hose” and “reliability” in sentence “Entries, whose reliability is above 99 and 95 % for bitopic and polytopic transmembrane proteins, respectively, were selected”.
- p. 8, the term “homologous partner’s structure” is not clear; please rephrase.
- p. 8 “filtering accuracy” is mentioned, without having been defined.
- p. 9 “ability to filter” is defined as “the ability to determine whether a sequence codes a TMP or a non-TMP. It may be more intuitive to name this parameter ”discriminating ability”.
- p. 14: Remove commas between “sequences” and “and”, as well as between “only” and “by” in the sentence “ Here, we established a new benchmark set, comprising sequences, and topologies of human TMPs only, by searching sequences homologous to the human sequences in the TOPDB database”
- p. 14: Replace: “have solved 3D structure” by “have a 3D structure”.
- p. 14: Remove comma between “human proteins“ and ”whose homologous partner”
- p. 15: Replace “by” by “of” in the phrase “is composed by three basic steps”
- p. 15: Replace sentence “Next, CCTOP makes a decision whether the investigated protein sequence codes a TMP or non-TMP” by “Next, CCTOP makes a decision as to whether the investigated protein sequence en codes a TMP or a non-TMP”
- p. 16: When describing the filtering step, the authors state that “we achieved higher accuracy by combining the tested methods, and reached 99 % both for sensitivity and specificity, respectively (Table 2)”. Where is the combined analysis shown in Table 2 ?
- p.17: Sentence “This should be the results that MEMSAT-SVM has the lowest prediction accuracy on 7 TMHs proteins using the Structure benchmark set” is not clear; please rephrase.
- p. 19: Change “monotonously decreasing” to “decreasing linearly”.

***Authors’ response:****We have amended the manuscript as suggested by the referee.*

**Quality of written English:** Needs some language corrections before being published

**Quality of Figures**: Acceptable.

## Additional files

Additional file 1:
**Prediction accuracies of tested methods.** Description: The prediction accuracies of all tested methods were measured using the structure benchmark set. MCC: Matthews Correlation Coeffitient, Acc_Tpg_: per protein topography accuracy, Acc_Tpl_: per protein topology accuracy, Comment: reason, why a given method was not suitable for inclusion in the final consensus method. Additional file 2:
**Title: Discrimination accuracy of majority decision algorithms.** Description: Description and additional information for the discrimination algorithm used int he CCTOP method. Additional file 3:
**Dependence of prediction accuracy on the number of used methods.** Description: Per protein topology prediction accuracies of the consensus algorithm are shown using the first n-best methods (red line) and the first n-worst methods (black line).

## Abbreviation

CCTOP: Constrained Consensus TOPology
HTP: Human Transmembrane Proteome
HSP: High-scoring Segment Pair
HMM: Hidden Markov Model
MCC: Matthews Correlation Coefficient
TMH: Transmembrane Helix
TMP: Transmembrane Protein

## References

[1] Kozma D, Simon I, Tusnády GE (2013). PDBTM: protein data bank of transmembrane proteins after 8 years. Nucleic Acids Res.

[2] Tusnády G, Dosztányi Z, Simon I (2005). PDB_TM: selection and membrane localization of transmembrane proteins in the protein data bank. Nucleic Acids Res.

[3] Tusnády G, Dosztányi Z, Simon I (2004). Transmembrane proteins in the protein data bank: identification and classification. Bioinformatics.

[4] Tusnady G, Kalmar L, Simon I (2008). TOPDB: topology data bank of transmembrane proteins. Nucleic Acids Res.

[5] Lander ES, Linton LM, Birren B, Nusbaum C, Zody MC, Baldwin J (2001). Initial sequencing and analysis of the human genome. Nature.

[6] Venter JC, Adams MD, Myers EW, Li PW, Mural RJ, Sutton GG (2001). The sequence of the human genome. Science.

[7] Hopf TA, Colwell LJ, Sheridan R, Rost B, Sander C, Marks DS (2012). Three-dimensional structures of membrane proteins from genomic sequencing. Cell.

[8] Käll L, Krogh A, Sonnhammer ELL (2004). A combined transmembrane topology and signal peptide prediction method. J Mol Biol.

[9] Käll L, Krogh A, Sonnhammer ELL (2007). Advantages of combined transmembrane topology and signal peptide prediction--the Phobius web server. Nucleic Acids Res.

[10] Bendtsen JD, Nielsen H, von Heijne G, Brunak S (2004). Improved prediction of signal peptides: SignalP 3.0. J Mol Biol.

[11] Petersen TN, Brunak S, von Heijne G, Nielsen H (2011). SignalP 4.0: discriminating signal peptides from transmembrane regions. Nat Methods.

[12] Yuan Z, Davis MJ, Zhang F, Teasdale RD (2003). Computational differentiation of N-terminal signal peptides and transmembrane helices. Biochem Biophys Res Commun.

[13] Gomi M, Akazawa F, Mitaku S (2000). SOSUIsignal: software system for prediction of signal peptide and membrane protein. Genome Informatics.

[14] Nielsen H, Engelbrecht J, Brunak S, von Heijne G (1997). Identification of prokaryotic and eukaryotic signal peptides and prediction of their cleavage sites. Protein Eng.

[15] Nielsen H, Brunak S, von Heijne G (1999). Machine learning approaches for the prediction of signal peptides and other protein sorting signals. Protein Eng.

[16] Reynolds SM, Käll L, Riffle ME, Bilmes JA, Noble WS (2008). Transmembrane topology and signal peptide prediction using dynamic bayesian networks. PLoS Comput Biol.

[17] Viklund H, Bernsel A, Skwark M, Elofsson A (2008). SPOCTOPUS: a combined predictor of signal peptides and membrane protein topology. Bioinformatics.

[18] Lao DM, Arai M, Ikeda M, Shimizu T (2002). The presence of signal peptide significantly affects transmembrane topology prediction. Bioinformatics.

[19] Tusnády GE, Simon I (2010). Topology prediction of helical transmembrane proteins: how far have we reached?. Curr Protein Pept Sci.

[20] De Miguel N, Lustig G, Twu O, Chattopadhyay A, Wohlschlegel JA, Johnson PJ (2010). Proteome analysis of the surface of Trichomonas vaginalis reveals novel proteins and strain-dependent differential expression. Mol Cell Proteomics MCP.

[21] Biller L, Matthiesen J, Kuehne V, Lotter H, Handal G, Nozaki T, Saito-Nakano Y, Schuemann M, Roeder T, Tannich E, Krause E, Bruchhaus I: The Cell Surface Proteome of Entamoeba histolytica. Mol Cell proteomics MCP. 2014;13:132-144. 10.1074/mcp.M113.031393 PMC3879609 24136294

[22] Gu B, Zhang J, Wang W, Mo L, Zhou Y, Chen L (2010). Global expression of cell surface proteins in embryonic stem cells. PLoS One.

[23] Niehage C, Steenblock C, Pursche T, Bornhäuser M, Corbeil D, Hoflack B (2011). The cell surface proteome of human mesenchymal stromal cells. PLoS One.

[24] Strassberger V, Gutbrodt KL, Krall N, Roesli C, Takizawa H, Manz MG, Fugmann T, Neri D: A comprehensive surface proteome analysis of myeloid leukemia cell lines for therapeutic antibody development. J Proteomics 2014;99:138-51. 10.1016/j.jprot.2014.01.022 24487095

[25] Tusnády GE, Simon I (2001). The HMMTOP transmembrane topology prediction server. Bioinformatics.

[26] Kim H, Melén K, von Heijne G (2003). Topology models for 37 Saccharomyces cerevisiae membrane proteins based on C-terminal reporter fusions and predictions. J Biol Chem.

[27] Rapp M, Drew D, Daley DO, Nilsson J, Carvalho T, Melén K (2004). Experimentally based topology models for E. coli inner membrane proteins. Protein Sci.

[28] Granseth E, Daley DO, Rapp M, Melén K, von Heijne G (2005). Experimentally constrained topology models for 51,208 bacterial inner membrane proteins. J Mol Biol.

[29] Krogh A, Larsson B, von Heijne G, Sonnhammer EL (2001). Predicting transmembrane protein topology with a hidden Markov model: application to complete genomes. J Mol Biol.

[30] Melén K, Krogh A, von Heijne G (2003). Reliability measures for membrane protein topology prediction algorithms. J Mol Biol.

[31] Tusnády GE, Kalmár L, Hegyi H, Tompa P, Simon I (2008). TOPDOM: database of domains and motifs with conservative location in transmembrane proteins. Bioinformatics.

[32] Bernsel A, von Heijne G (2005). Improved membrane protein topology prediction by domain assignments. Protein Sci.

[33] Nilsson J, Persson B, von Heijne G (2000). Consensus predictions of membrane protein topology. FEBS Lett.

[34] Käll L, Sonnhammer ELL (2002). Reliability of transmembrane predictions in whole-genome data. FEBS Lett.

[35] Wu CH, Apweiler R, Bairoch A, Natale DA, Barker WC, Boeckmann B (2006). The universal protein resource (UniProt): an expanding universe of protein information. Nucleic Acids Res.

[36] Tusnády GE, Dosztányi Z, Simon I (2005). TMDET: web server for detecting transmembrane regions of proteins by using their 3D coordinates. Bioinformatics.

[37] Kim H, Melén K, Osterberg M, von Heijne G (2006). A global topology map of the Saccharomyces cerevisiae membrane proteome. Proc Natl Acad Sci U S A.

[38] Li W, Godzik A (2006). Cd-hit: a fast program for clustering and comparing large sets of protein or nucleotide sequences. Bioinformatics.

[39] Huang Y, Niu B, Gao Y, Fu L, Li W (2010). CD-HIT Suite: a web server for clustering and comparing biological sequences. Bioinformatics.

[40] Griep S, Hobohm U (2010). PDBselect 1992–2009 and PDBfilter-select. Nucleic Acids Res.

[41] Nugent T, Jones DT (2010). Predicting transmembrane helix packing arrangements using residue contacts and a force-directed algorithm. PLoS Comput Biol.

[42] Viklund H, Elofsson A (2008). OCTOPUS: improving topology prediction by two-track ANN-based preference scores and an extended topological grammar. Bioinformatics.

[43] Viklund H, Elofsson A (2004). Best alpha-helical transmembrane protein topology predictions are achieved using hidden Markov models and evolutionary information. Protein Sci.

[44] Bernsel A, Viklund H, Falk J, Lindahl E, von Heijne G, Elofsson A (2008). Prediction of membrane-protein topology from first principles. Proc Natl Acad Sci U S A.

[45] Sonnhammer EL, von Heijne G, Krogh A (1998). A hidden Markov model for predicting transmembrane helices in protein sequences. Proc Int Conf Intell Syst Mol Biol.

[46] Tusnády GE, Simon I (1998). Principles governing amino acid composition of integral membrane proteins: application to topology prediction. J Mol Biol.

[47] Shen H, Chou JJ (2008). MemBrain: improving the accuracy of predicting transmembrane helices. PLoS One.

[48] Nugent T, Jones DT (2009). Transmembrane protein topology prediction using support vector machines. BMC Bioinformatics.

[49] Reddy A, Cho J, Ling S, Reddy V, Shlykov M, Saier MH (2014). Reliability of nine programs of topological predictions and their application to integral membrane channel and carrier proteins. J Mol Microbiol Biotechnol.

[50] Bagos PG, Liakopoulos TD, Hamodrakas SJ (2006). Algorithms for incorporating prior topological information in HMMs: application to transmembrane proteins. BMC Bioinformatics.

[51] Rabiner LR (1989). A tutorial on hidden Markov models and selected applications in speech recognition. Proc IEEE.

[52] Ivankov DN, Payne SH, Galperin MY, Bonissone S, Pevzner PA, Frishman D (2013). How many signal peptides are there in bacteria?. Environ Microbiol.

[53] Pieper U, Schlessinger A, Kloppmann E, Chang GA, Chou JJ, Dumont ME (2013). Coordinating the impact of structural genomics on the human α-helical transmembrane proteome. Nat Struct Mol Biol.

[54] Kim M-S, Yi G-S (2013). HMPAS: human membrane protein analysis system. Proteome Sci.

[55] VENNY. An interactive tool for comparing lists with Venn Diagrams [http://bioinfogp.cnb.csic.es/tools/venny/index.html].

[56] Wt, C++ Web Toolkit [http://www.webtoolkit.eu/wt]

[57] Kozma D, Simon I, Tusnády GE (2012). CMWeb: an interactive on-line tool for analysing residue-residue contacts and contact prediction methods. Nucleic Acids Res.

[58] Wang J, Duncan D, Shi Z, Zhang B (2013). WEB-based GEne SeT AnaLysis toolkit (WebGestalt): update 2013. Nucleic Acids Res.

