# Supplementary material for: The human transmembrane proteome
Source: Biol Direct. 2015 May 28;10:31. doi: 10.1186/s13062-015-0061-x (PMC4445273; doi:10.1186/s13062-015-0061-x)
Supplement: Additional file 1: — Prediction accuracies of tested methods. Description: The prediction accuracies of all tested methods were measured using the structure benchmark set. MCC: Matthews Correlation Coeffitient, AccTpg: per protein topography accuracy, AccTpl: per protein topology accuracy, Comment: reason, why a given method was not suitable for inclusion in the final consensus method. [file 13062_2015_61_MOESM1_ESM.doc]

| **Name** | **Sensitivity** | **Specificity** | **MCC** | **Acc_Tpg_** | **Acc_Tpl_** | **Comments and references** |
| --- | --- | --- | --- | --- | --- | --- |
| **DAS** | 0.88 | 0.93 | 0.90 | 55 | - | Low accuracies, has not been selected [*] |
| **HMMTOP** | 0.97 | 0.97 | 0.97 | 84 | 81 | Selected, runs locally [25,45] |
| **MemBrain** | 0.99 | 0.94 | 0.96 | 76 | - | Selected, runs via internet [46] |
| **MEMSAT-SVM** | 0.96 | 0.99 | 0.97 | 80 | 68 | Selected, runs locally [47] |
| **Octopus** | 0.97 | 0.98 | 0.97 | 83 | 82 | Selected, runs locally [42] |
| **Philius** | 0.96 | 0.98 | 0.97 | 81 | 74 | Selected, runs locally [16] |
| **Phobius** | 0.97 | 0.97 | 0.97 | 79 | 77 | Selected, runs locally [9] |
| **Pro** | 0.90 | 0.98 | 0.94 | 66 | 51 | Selected, runs locally [48] |
| **Prodiv** | 0.97 | 0.97 | 0.97 | 82 | 67 | Selected, runs locally [48] |
| **Scampi-Single** | 0.91 | 0.92 | 0.92 | 49 | 46 | Low accuracies, has not been selected, runs locally [43] |
| **Scampi-MSA** | 0.99 | 0.98 | 0.98 | 89 | 88 | Selected, runs locally [43] |
| **SOSUI** | 0.90 | 0.96 | 0.93 | 60 | 0 | Server is unstable, has not been selected [**] |
| **TMHMM** | 0.94 | 0.99 | 0.96 | 76 | 70 | Selected, runs locally [29,44] |
| **Valpred** | 0.91 | 0.84 | 0.88 | 40 | - | Low accuracies, has not been selected [***] |

* Cserzo M, Wallin E, Simon I, von Heijne G, Elofsson A: **Prediction of transmembrane alpha-helices in prokaryotic membrane proteins: the dense alignment surface method**. *Protein Eng Des Sel* 1997, **10**:673–676.

** Hirokawa T, Boon-Chieng S, Mitaku S: **SOSUI: classification and secondary structure prediction system for membrane proteins**. *Bioinformatics* 1998, **14**:378–379.

*** Dastmalchi S, Morris MB, Church WB: **Modeling of the structural features of integral-membrane proteins reverse-environment prediction of integral membrane protein structure (REPIMPS).** *Protein Sci* 2001, **10**:1529–38.
