# Supplementary material for: The human transmembrane proteome
Source: Biol Direct. 2015 May 28;10:31. doi: 10.1186/s13062-015-0061-x (PMC4445273; doi:10.1186/s13062-015-0061-x)
Supplement: Additional file 2: — Title: Discrimination accuracy of majority decision algorithms. Description: Description and additional information for the discrimination algorithm used int he CCTOP method. [file 13062_2015_61_MOESM2_ESM.doc]

**Discrimination between transmembrane and non transmembrane proteins**

We have selected individual methods with Matthews correlation coefficients above 0.93 on the filtering benchmark set (see Table 2 in the main text). By using all of them in a majority decision algorithm the false negative (FN) and false positive (FP) ratios decreased, but the true positive (TP) ratio decreased as well. Therefore, we got better results when we utilized only three out of the selected four methods. The following table shows the results of all triplet combinations of the selected four methods. As it can be seen, the highest accuracy could be reached if the three methods were TMHMM, Scampi and Phobius.

| **Methods** | | | | **TP** | **TN** | **FP** | **FN** | **Sensitivity** | **Specificity** | **MCC** |
| --- | --- | --- | --- | --- | --- | --- | --- | --- | --- | --- |
| **Philius** | Phobius | Scampi | TMHMM |
| **+** | + | + |  | 465 | 1,403 | 19 | 9 | 0.98 | 0.99 | 0.96 |
| **+** | + |  | + | 459 | 1,403 | 19 | 15 | 0.97 | 0.99 | 0.95 |
| **+** |  | + | + | 465 | 1,403 | 19 | 9 | 0.98 | 0.99 | 0.96 |
|  | + | + | + | 467 | 1,401 | 21 | 7 | 0.99 | 0.99 | 0.96 |
